# Drugs that target pathogen public goods are robust against evolved drug resistance

**DOI:** 10.1111/j.1752-4571.2012.00254.x

**Published:** 2012-11

**Authors:** John W Pepper

**Affiliations:** 1 Division of Cancer Prevention, National Cancer InstituteBethesda, MD, USA; 2 Santa Fe InstituteSanta Fe, NM, USA

**Keywords:** biomedicine, cancer medicine, contemporary evolution, disease biology, evolutionary medicine, evolutionary theory, experimental evolution, microbial biology, natural selection

## Abstract

Pathogen drug resistance is a central problem in medicine and public health. It arises through somatic evolution, by mutation and selection among pathogen cells within a host. Here, we examine the hypothesis that evolution of drug resistance could be reduced by developing drugs that target the secreted metabolites produced by pathogen cells instead of directly targeting the cells themselves. Using an agent-based computational model of an evolving population of pathogen cells, we test this hypothesis and find support for it. We also use our model to explain this effect within the framework of standard evolutionary theory. We find that in our model, the drugs most robust against evolved drug resistance are those that target the most widely shared external products, or ‘public goods’, of pathogen cells. We also show that these drugs exert a weak selective pressure for resistance because they create only a weak correlation between drug resistance and cell fitness. The same principles apply to design of vaccines that are robust against vaccine escape. Because our theoretical results have crucial practical implications, they should be tested by empirical experiments.

## Introduction

Pathogen drug resistance is a central problem in medicine and public health. It is central both to infectious disease (Fong and Drlica 2003) and also to cancer medicine. A cancer is a population of endogenous pathogen cells, which often evolves resistance to the drugs used to treat it. Consequently, the development of drug resistance within a patient is a central problem in cancer biology (Pepper 2008; Moscow et al. 2003; O’Connor et al. 2007; Pepper et al. 2009). Regardless of their origin, pathogen cells acquire drug resistance through somatic (within host) mutation and selection. Somatic selection is the preferential survival and proliferation of cells with mutations allowing them to resist a therapeutic drug. Those cells then pass that resistance to an expanding lineage of progeny cells (Pepper 2008).

Because drug resistance arises though somatic selection and evolution, understanding this process is crucial to addressing the problem. It was recently proposed that evolution of drug resistance could be reduced by developing drugs that do not directly target pathogen cells themselves but instead target the secreted metabolites, or ‘public goods’ compounds, that they use to modify their microenvironment to their advantage (Pepper 2008). To examine this hypothesis, we use simulations based on an agent-based computational model.

This study examines the implications for drug and vaccine design of the theory for public goods evolution developed by (Driscoll and Pepper 2010). This theory was based on the physics of diffusion, along with some basic assumptions about fitness effects of diffusible cell products. It has not previously been closely integrated with standard evolutionary theory. Here, we pursue this integration through the Price equation, which represents the change per generation in the mean value of a trait as the covariance between trait value and fitness (Price 1970, 1972; Hamilton 1975). This covariance is factored into three components representing standard deviation of the trait value of interest, standard deviation of fitness, and the Pearson’s correlation between trait value and fitness (Price 1970; Eqn. 1). If indeed selection for drug resistance is weaker on drugs that target public goods than on drugs that target cell-intrinsic traits, that outcome must be due to a lower value of one or more of these three components of selection. To test this hypothesis, we used an *in silico* evolutionary system (Adami and Wilke 2004; Pennock 2007) in which all relevant variables could be quantified and controlled.

The potential molecular targets of therapeutic drugs fall on a spectrum from molecules that are strictly ‘private’, because they are intrinsic to the cell that produces them, to those that at are more ‘public’ in their fitness effects, because they are shared among cells other than their producer. The strength of selection on cells to produce and maintain a molecular trait depends strongly on where the specific molecule lies on this spectrum from private to public. An earlier study quantified the spectrum from private to public molecules using a ‘transfer coefficient’ that combines the diffusion coefficient of the molecule and the diffusion distance among cells to quantify the transferability of the molecule among cells (Driscoll and Pepper 2010).

In that earlier work (Driscoll and Pepper 2010), and its extension here, we assume there is some metabolic cost to the production of any molecular product. Only producer cells pay this cost, while neighboring cells can enjoy the benefits of transferable (diffusible) beneficial products without paying the cost of production. When benefits of external goods are shared but costs are not, production is selectively favored only under restrictive conditions. In particular, mathematical models showed that production of more private beneficial products, including cell-intrinsic molecules, is more robustly favored than is the production of more widely shared or ‘public’ beneficial products (Driscoll and Pepper 2010). That result has important implications for the evolution of drug resistance, and we seek to replicate it here. We predict that when a drug interferes with a molecular target benefiting pathogen cells, any cell producing a mutant form of the product that is impervious to the drug will be more strongly favored if the molecular target is more private than if it is widely shared. Consequently, we also predict that resistance will evolve more rapidly against drugs targeting ‘private’ molecules and more slowly against drugs targeting ‘public’ molecules.

Because social evolution involves feedbacks, spatial heterogeneity, and other nonlinear effects, linear analytical models such as those developed previously (Driscoll and Pepper 2010) can provide only simplified representations of expected outcomes. We can improve on their predictive power by building agent-based computational models that explicitly represent each cell as a computational agent, and include the spatial relationships among cells and their external products. Using such a computational model, we performed simulation experiments as preliminary tests of the hypothesis that drugs targeting shared cellular products will reduce the evolution of pathogen drug resistance, as compared to drugs targeting cell-intrinsic molecules.

## Methods

In our computational model, both time and space were discrete, with space represented as a 2D grid of locations, each representing the distinct microenvironment influenced by a cell (approximately 1 square micron), and characterized by local concentration of a potentially soluble target molecule produced by pathogen cells. This solute diffused through space down its concentration gradient. As a reference diffusion coefficient, we used 1.56 × 10^−6^ cm^2^ s^−1^. While the model was not tuned to a specific molecule, this diffusion rate is typical of biologically relevant small molecules (Stewart 2003). As experimental treatments, we ran simulation experiments using drug targeting molecules with 0, 1, and 2 times this reference diffusion coefficient. Each pathogen cell was represented as a computational agent characterized by its vitality *v* (propensity to survive and divide) and by its binary resistance or sensitivity to the drug. Pathogen cells were capable of mitosis (with cell heritability of resistance state) and of death. The stochastic probability of each of these outcomes per time step was determined by the cell’s vitality, which was a function of its access to beneficial cell product in its microenvironment, and by its level of drug resistance (which was inherited during division). Cells with lower vitality were more likely to die (probability = 1−*v*). Surviving cells then reproduced with a fixed probability (of 90%) if an adjacent location was unoccupied. Each daughter cell inherited half of the vitality of the parent cell, and each mutated with a fixed probability of 10^−3^ between the drug-sensitive and drug-resistant states. Upon division, one daughter cell moved to inhabit the adjacent, vacant location, while the other replaced its parent. These simple physical assumptions of localized reproduction and localized diffusion made indirect fitness effects a natural and inescapable consequence, but we did not explicitly quantify the strength of direct versus indirect fitness effects in this study.

Our experiments were conducted on a 51 × 51 rectangular grid containing 2601 discrete locations. Thus, the population size of pathogen cells was also capped at a maximum of 2601.

### Model scheduling

**Figure 1:**
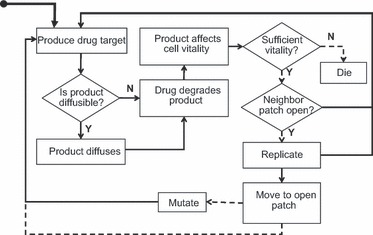
A graphical representation of the flow of events for each cell during each time step within the computational model. Dashed lines denote stochastic steps not always taken.

Simulations progressed in discrete time steps corresponding to 40 min, the approximate generation time of *Pseudomonas aeruginosa* growing at the body temperature of a human host (El’Garch et al. 2007). The order in which cells were updated was randomized within each time step. During updating, each cell released its molecular product into its own microenvironment. The product of nonresistant cells was then degraded according to drug concentration. The cell then updated its vitality as a function of local concentration of the beneficial product. Next, the cell had a stochastic chance of dying, dependent on is current vitality. If it survived, it had a stochastic chance of dividing. See Fig. 1 for a flowchart representation of this scheduling.

Data collection began as soon as the first stochastic mutation in drug resistance arose in the population, creating genetic variation for selection to act upon. The simulation run then continued for another 1000 time steps before summary statistics were compiled.

### Instance variables of cells

#### Vitality

The vitality change in an individual cell in each time step depended upon the concentration of the external product experienced by the cell and also on the cell’s previous fitness value. The fitness change for a single time step followed a logistic function: *Δv* = [*p*] (1−v), where v is vitality and [*p*] is local product concentration. Survival probability per time step was directly proportional to vitality. While realized fitness (reproduction) of an individual cell was stochastic and was also affected by space availability, our term ‘vitality’ refers to a variable indicating relative fitness based on the biochemical status of the cell.

### Instance variables of locations

Each location in space carried a specified concentration of both drug-sensitive and drug-resistant cell products. The flux of each product between two adjacent locations per time step was a function of the concentration gradient between them and the specified transfer coefficient. We used values of 0, 0.5, and 1.0 times the reference diffusion coefficient of 3.12 × 10^−6^ cm^2^ s^−1^. These correspond to a drug target that is cell-intrinsic and not shared (T = 0) and to two biologically plausible levels of transferability in the molecule used as a drug target.

### Initialization

At the outset of each simulation run, each location contained a single drug-sensitive cell with vitality (*v*) randomly drawn from a uniform distribution between 0 and 1.

### System parameters

The experimentally manipulated variable of interest was the transfer coefficient of the molecule targeted by the drug. Because lower drug dosage is conducive to the evolution of drug resistance, we conducted each virtual experiment with a range of doses, including the reference 100%‘full dose’ with maximal effect, as well as 75% and 50% of this dose.

## Results

**Figure 2:**
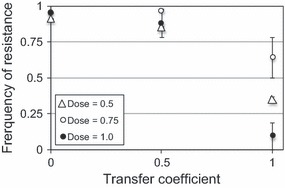
Simulation results: Frequency of the drug resistance mutation averaged over last 1000 cell generations. Each marker represents the mean for a different drug concentration. High transfer coefficients correspond to drug targets that are more ‘public’ or more widely shared among cells. A value of zero corresponds to a cell-intrinsic drug target. Markers show the mean, and bars show the standard error across 10 simulation runs with different seed values for the pseudorandom number generator.

In the simulation results, the final frequency of drug resistance among pathogen cells (after evolution) was lower when the drug targeted a pathogen public good, rather than an intrinsic cell traits (Fig. 2). For each drug dosage tested, the final frequency of drug-resistant cells was significantly lower for drugs targeting the most ‘public’ molecule (transfer coefficient = 1) than for drugs targeting less-shared molecules. Comparing a transfer coefficient value of 1 vs either 0.5 or 0 (with drug dose set at 1.0), the mean frequency of drug-resistant cells was significantly lower when the drug target was highly shared (one-tailed *t*-test with unequal variances, *P* < 0.0001 for each comparison).

**Figure 3:**
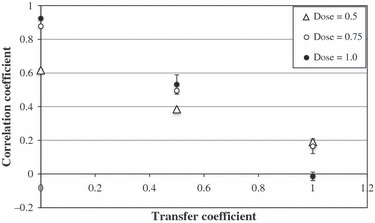
Simulation results: Correlation across cells between drug resistance and cell fitness (vitality). Correlation values were averaged over 1000 cell generations. A value of zero for transfer coefficient represents a cell-intrinsic drug target. Markers show means, and bars show standard errors across 10 simulation runs with different seed values for the pseudorandom number generator. Results are shown for three different drug concentrations.

To clarify the mechanistic basis for this result, we quantified each of the three factors determining the rate of evolution of drug resistance. We found that for a drug targeting a more shared or ‘public’ molecule, variation among cells was higher, both in their in their level of resistance and in their vitality, or fitness (results not shown). However, selection was weaker despite this higher variance. This was because when the transfer coefficient was higher, the correlation between resistance and cell vitality was substantially lower (Fig. 3). For each of three comparisons between transfer coefficient values of 0, 0.5, and 1.0 (with drug dose set at 1.0), the mean correlation coefficient was significantly lower when the transfer coefficient was higher (one-tailed *t*-test with unequal variances, *P* < 0.0001 for each comparison).

**Figure 4:**
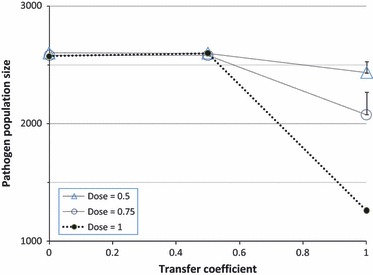
Simulation results: Mean size of total pathogen population, averaged over last 1000 cell generations. A value of zero for transfer coefficient represents a cell-intrinsic drug target. Markers show means, and bars show standard errors across 10 simulation runs with different seed values for the pseudorandom number generator. (Some error bars are too small to be visible.) Results are shown for three different drug concentrations.

When pathogens were treated with a drug targeting a shared rather than a cell-intrinsic molecule, the reduced evolution of drug resistance resulted in a smaller final population of pathogen cells (Fig. 4). Comparing a drug target with a transfer coefficient of 1 against values of either 0 or 0.5 (with a drug dose of 1.0), the final pathogen population was significantly smaller when the drug targeted a more ‘public’ molecule (one-tailed *t*-test with unequal variances, *P* < 0.0001 for each comparison).

## Discussion

Our results support the proposal that evolved drug resistance in pathogens can be reduced by using agents directed against external products rather than cell-intrinsic molecules. Furthermore, as there is a spectrum from private to public external products (Pepper 2008), the most robust targets for therapeutic intervention are those that are most widely shared (as quantified by a high transfer coefficient).

The reduced tendency for evolution of resistance against such drugs is attributable to the reduced correlation between resistance and cellular fitness (Fig. 3). Our focus here on cell-level fitness contrasts with earlier theory that used the approach of multilevel selection and explicitly recognized the role of selection among trait groups defined by their shared microenvironment of diffusible products (Andre and Godelle 2005; Pepper 2008). These two approaches are not mutually exclusive, and it is expected that they should arrive at the same conclusions. It is well understood in social evolution theory that trait-group selection is not a distinct process from either selection for inclusive fitness or selection for the neighbor-modulated fitness considered here. Rather, it is simply an alternative accounting system (West et al. 2007). Partitioning selection into direct versus indirect (neighbor-modulated) components is neither more nor less correct than partitioning it into within-group versus between-group components. Indeed, it is well established that the two mathematical frameworks are formally equivalent and interconvertible (Hamilton 1975; Wade 1985; Pepper 2007). Averaging cell fitness across trait groups, as we do here, does not imply a different process from trait-group selection, nor should it lead to different conclusions if performed correctly (Okasha 2004).

The current results are consistent with those of a mathematical model that focused on the evolution of drug resistance in bacteria and found an advantage for drugs blocking bacterial cooperation, especially communication (Andre and Godelle 2005). They are also consistent with those of more general mathematical models (Pepper 2008).

Our current results have rather wide applicability to pathogens, including both infectious diseases and cancers. Our conclusions will be valid in any case where the key model assumptions are met: that individual cells vary in drug resistance and that this trait is heritable; that available drugs do not achieve perfectly efficacy, killing all cells instantly; and that some fitness-enhancing molecules produced by pathogen cells have shared fitness benefits. Examples of such public goods produced by bacterial pathogens include quorum-sensing molecules, siderophores, extracellular polymeric matrix, and exotoxins; for cancer cells, examples include growth and invasion factors, angiogenesis factors, and immune suppression factors (Pepper 2008). We note that this study addressed only robustness against evolved resistance and that drug efficacy is a separate consideration.

We have mostly focused on the potential for targeting public goods as a way to prevent the future evolution of resistance to new drugs. However, it may also be possible to robustly block existing mechanisms of resistance to older antibacterials, thereby bringing them back into effectiveness. It was recently shown that bacterial resistance to antibiotics can be mediated as a population-level trait by the secreted signaling molecule indole, which protects any bacterial cells receiving it against the effects of antibiotics (Lee et al. 2010). Because indole is a shared cellular product, we predict that therapeutics targeting it not only could block antibiotic resistance in the short term but also would themselves be robust against evolved resistance in the longer term.

The principles revealed here apply to the development of vaccines as well as drugs. The same principles govern the adaptive evolutionary response of microbes to any molecularly targeted fitness challenge, whether pharmacological or immunological. Thus, to avoid immune escape by pathogens, any public goods they rely on should be preferred targets for the design of vaccines as well as drugs (Pepper 2008).
